# Usefulness of driver’s eye movement measurement to detect potential risks under combined conditions of taking second-generation antihistamines and calling tasks

**DOI:** 10.1186/s40780-024-00383-5

**Published:** 2024-10-02

**Authors:** Atsunobu Sagara, Akihito Nagahama, Hayato Aki, Hiroki Yoshimura, Makoto Hiraide, Takatsune Shimizu, Motohiko Sano, Tetsuro Yumoto, Tomoo Hosoe, Kenji Tanaka

**Affiliations:** 1 School of Pharmacy and Pharmaceutical Sciences, Hoshi University, 2-4-41 Ebara, Shinagawa-Ku, Tokyo, Japan; 2 Graduate School of Informatics and Engineering, The University of Electro-Communications, 1-5-1, Chofugaoka, Chofu-Shi, Tokyo, Japan

**Keywords:** Driving simulator, Second-generation antihistamine, Calling task, Eye movement

## Abstract

**Background:**

Concerns persist regarding the potential reduction in driving performance due to taking second-generation antihistamines or performing hands-free calling. Previous studies have indicated a potential risk to driving performance under an emergency event when these two factors are combined, whereas a non-emergency event was operated effectively. Currently, there is a lack of a discriminative index capable of detecting the potential risks of driving performance impairment. This study aims to investigate the relationship between driving performance and eye movements under combined conditions of taking second-generation antihistamines and a calling task, and to assess the usefulness of eye movement measurements as a discriminative index for detecting potential risks of driving performance impairment.

**Methods:**

Participants engaged in a simulated driving task, which included a calling task, both under taking or not taking second-generation antihistamines. Driving performance and eye movements were monitored during both emergency and non-emergency events, assessing their correlation between driving performance and eye movements. The study further evaluated the usefulness of eye movement as a discriminative index for potential driving impairment risk through receiver operating characteristic (ROC) analysis.

**Results:**

In the case of a non-emergency event, no correlation was observed between driving performance and eye movement under the combined conditions. Conversely, a correlation was observed during an emergency event. The ROC analysis, conducted to assess the discriminative index capability of eye movements in detecting the potential risk of driving performance impairment, demonstrated a high discriminative power, with an area under the curve of 0.833.

**Conclusions:**

The findings of this study show the correlation between driving performance and eye movements under the concurrent influence of second-generation antihistamines and a calling task, suggesting the usefulness of eye movement measurement as a discriminant index for detecting potential risks of driving performance impairment.

## Background

Since the 1990s, there has been a significant increase in the prevalence of allergic rhinitis [1], with second-generation antihistamines being the recommended primary treatment [2]. Although second-generation antihistamines have reduced sedative effects compared to their first-generation counterparts, they can still negatively affect the ability to drive a motor vehicle [3]. At the same time, improvements in mobile phone technology have made it easier to take calls via hands-free devices while driving, a practice that has been linked to poorer driving outcomes [4]. Consequently, the concurrent use of second-generation antihistamines and hands-free calling poses a potential hazard for drivers. Although the specific effects of this combination on driving were initially uncertain, recent findings suggest a notable degradation in driving capabilities during emergencies [5]. Despite the need to detect the potential risk of impaired driving under these combined conditions, no discriminative index currently exists. Previous studies have established a correlation between dangerous driving and eye movements [6, 7], suggesting that measuring drivers’ eye movements, particularly in the horizontal direction, could potentially detect these risks [8].

This study aims to investigate the correlation between driving performance and eye movements under combined conditions of taking second-generation antihistamines and performing hands-free calling, and to assess the usefulness of eye movement measurements as a discriminative index for detecting potential risks of driving performance impairment.

## Methods

### Participants

The study recruited participants who met specific inclusion criteria: they were seasonal users of one of the following second-generation antihistamines—fexofenadine, bepotastine, levocetirizine, or ketotifen—due to hay fever, without being regular users throughout the year. Additionally, they should not have had any prior experience with driving simulators. Invitation to participate was extended through application forms distributed to individuals without any vested interest in the research. Exclusion criteria for the study included people taking medicines other than those in the study and people with ophthalmological conditions. Prior to eye movement measurements, it was confirmed that no participant had ophthalmological conditions that could affect their visual field. Informed consent was obtained from all participants after they were fully briefed on the study’s objectives.

### Experimental

**Fig. 1:**
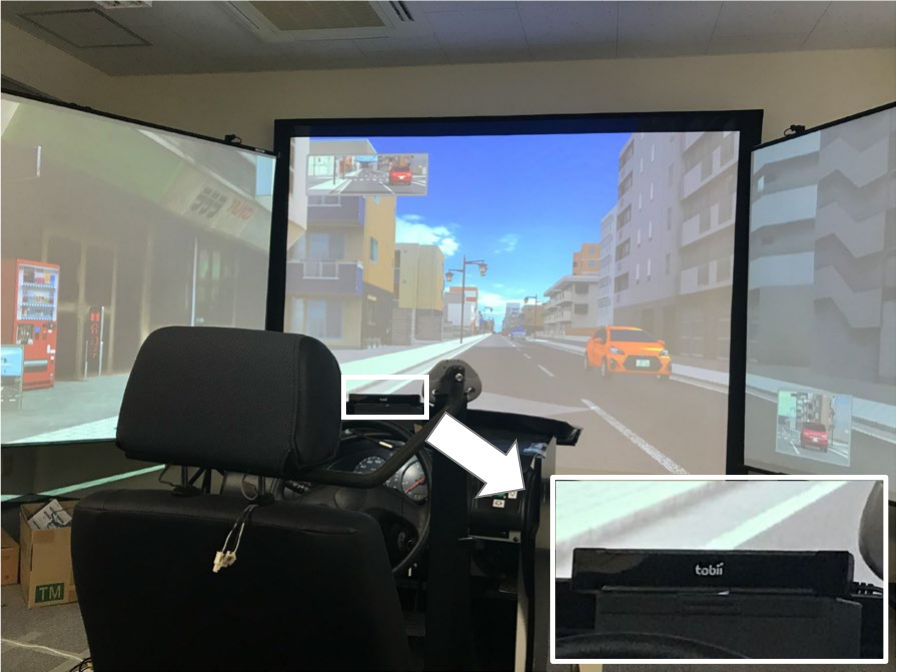
Experimental setup showing the main view with an inset detail of the eye tracker in the lower right corner

**Fig. 2:**
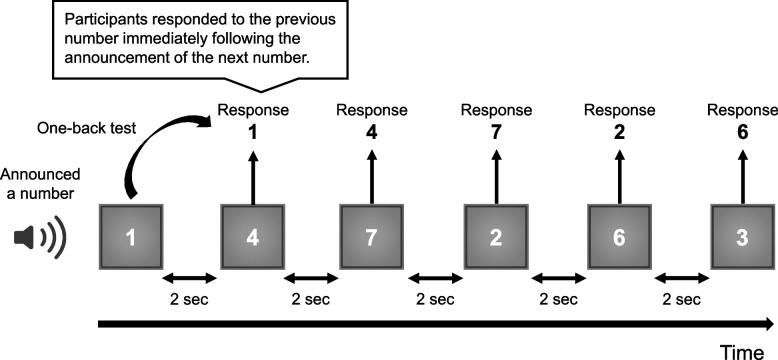
Image of one-back task as a calling task

**Fig. 3:**
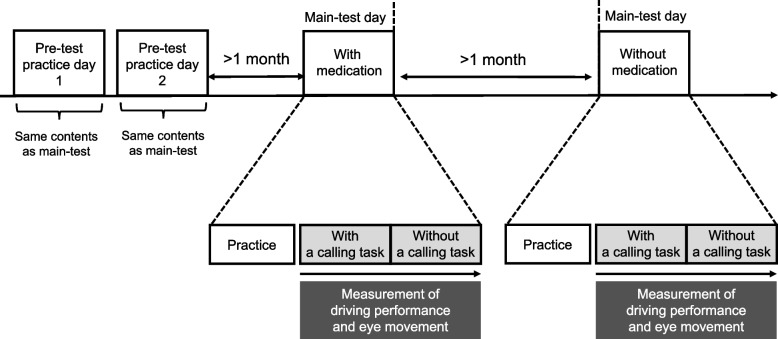
Experimental schedule of this study

For the experiment, a Mitsubishi Precision in-vehicle driving simulator was used. Participants engaged with the simulation through a large screen, 1.9 m in height and 3.1 m in width, which displayed the driving environment. Participants operated the simulator using realistic vehicle controls, such as the steering wheel, accelerator, and brake pedals, providing an experience akin to driving a real car (Fig. 1) The participants undertook a one-back task [9] as a calling task (Fig. 2) [10]. An audio file, pre-recorded with single-digit numbers spoken randomly every two seconds, was played during the driving simulation. Participants were tasked with responding to the number previously announced immediately following the announcement of the next number. To reduce the effects of familiarity with the task, participants underwent two days of preliminary training on the driving simulator and the one-back task, identical to the setup of the main experiment (Fig. 3). They used the simulator on different days to evaluate their driving performance and eye movements under taking and not taking medications. To account for potential fatigue from operating the simulator, the calling task was conducted at the beginning of the main test day, followed by the main test without the calling task. During the medication phase of the main study, participants consistently took each medication for at least seven days beforehand to achieve a stable concentration in their bodies. Following this phase, a washout period of at least one month was implemented before proceeding with the main study without medication.

### Evaluation of events and eye movements

**Fig. 4:**
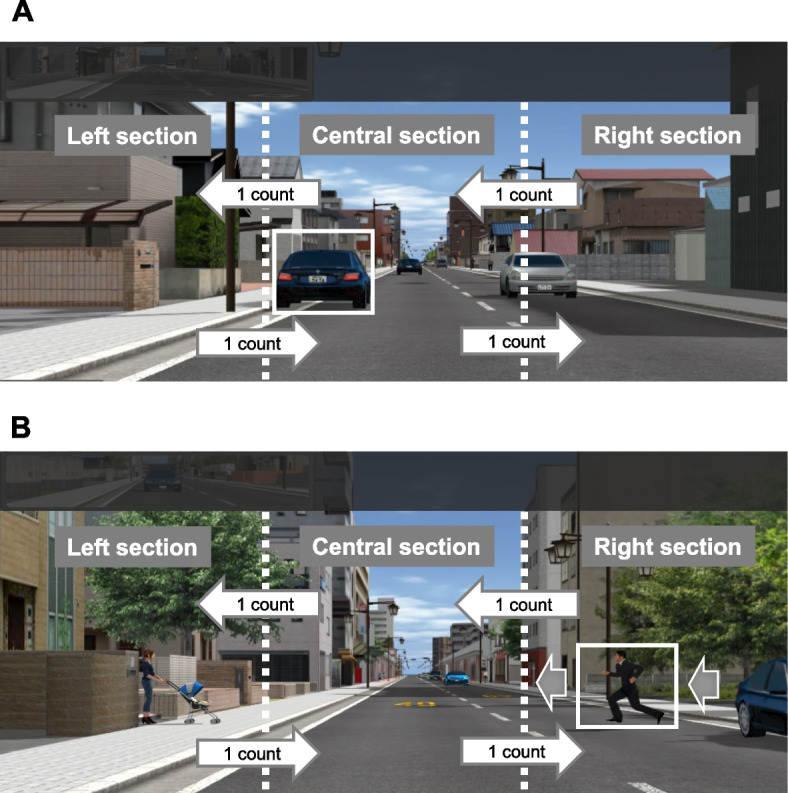
Image of a non-emergency event (**A**), and an emergency event (**B**) in the driving simulator

The assessment of driving performance was structured around two distinct events. For a non-emergency event, the stopped vehicle was positioned within the lane of the participant's vehicle. The measure was the smallest distance maintained between the stopped vehicle and the participant's vehicle while passing (Fig. 4A). In an emergency event, a pedestrian unexpectedly emerged from behind the stationary vehicle as the participant's vehicle approached within 30 m of it from the opposite lane. The measure was the smallest distance between the pedestrian and the participant’s vehicle (Fig. 4B). Both events demonstrate that as the distance between the object and the participant's vehicle decreases, driving performance deteriorates.

Eye movements including saccades were monitored using a Tobii X2 Eye Tracker, a non-intrusive device positioned on the dashboard to avoid impeding the driver’s view (Fig. 1). Horizontal eye movements were analyzed by dividing the front screen into three zones: left, center, and right. A transition from one zone to another, except for the screen’s upper area which includes the rear-view mirror, was recorded as a single movement (Fig. 4A and B). Eye movement data were collected from a distance of 100 m prior to predefined events up to the occurrence of the event itself. The ratio of eye movements was then calculated for each participant, defined as the number of eye movements by an individual relative to the maximum number of eye movements recorded across all participants in the study.

### Analysis

Scatter plots were generated depicting the correlation between the smallest distance in a non-emergency event or an emergency event, and the ratio of eye movements. Spearman　rank correlation coefficient was calculated to determine the strength of these relationships. Additionally, the receiver operating characteristic (ROC) curve was plotted, and the area under the curve (AUC) values were computed to evaluate the usefulness of eye movement measurements as a discriminative index for detecting potential risks of driving performance impairment [11]. All analyses were conducted using JMP17 Pro software (SAS Institute, CA) A power analysis was conducted beforehand using G-power. With an effect size of 0.6, an alpha error of 0.05, and a power of 0.8, the required total sample size was calculated to be 17 participants. Therefore, 19 participants were included in this study. Additionally, post-hoc analysis revealed that Fig. 6D had a power of 0.934.

## Results

The selection process yielded 19 hay fever sufferers aged between 20 and 50 years (mean age = 34.9 years; SD = 8.07, comprising 17 males and 2 females). The distribution of antihistamine usage among participants was as follows: fexofenadine (5), bepotastine (5), levocetirizine (5), and ketotifen (4). All participants possessed a valid driving license, with an average driving experience of 15.0 years (SD = 7.76), and none were on any other medication that could influence the study’s outcomes.

**Fig. 5:**
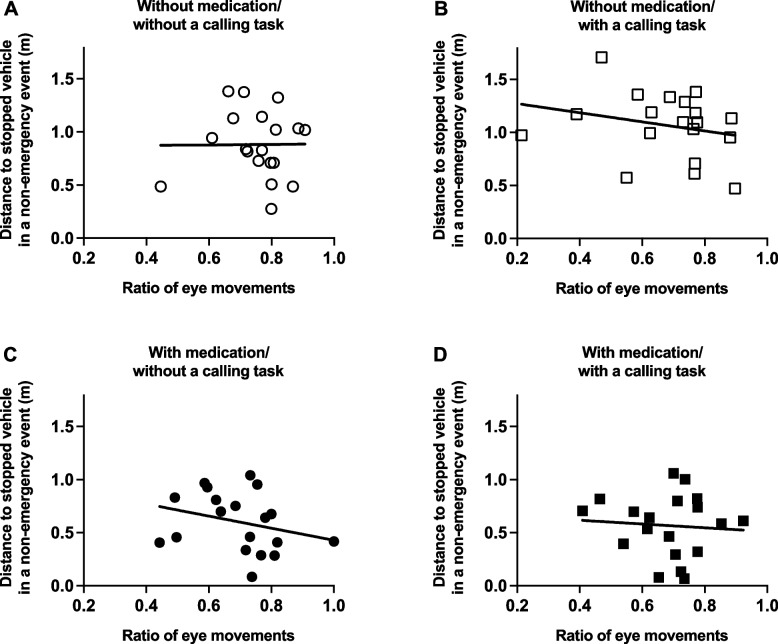
In a non-emergency event, a correlation between the smallest distance when the participant’s vehicle overtook the stopped vehicle and the ratio of eye movements under conditions without medication/without a calling task (**A**), without medication/with a calling task (**B**), with medication/without a calling task (**C**), and with medication/with a calling task (**D**)

**Fig. 6:**
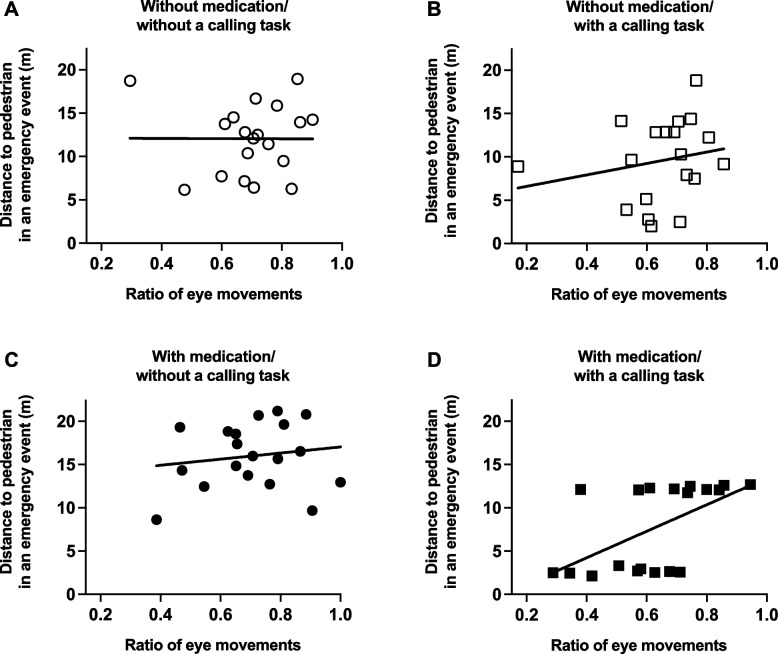
In an emergency event, a correlation between the smallest distance from the participant’s vehicle to pedestrian and the ratio of eye movements under conditions without medication/without a calling task (**A**), without medication/with a calling task (**B**), with medication/without a calling task (**C**), and with medication/with a calling task (**D**)

In the context of a non-emergency event with/without medication and with/without a calling task, no correlation was observed between the smallest distance when the participant’s vehicle overtook the stopped vehicle and the ratio of eye movements (without medication/without a calling task, rs = -0.117, p = 0.634, Fig. 5A; without medication/with a calling task, rs = -0.361, *p* = 0.128, Fig. 5B; with medication/without a calling task, rs = -0.242, *p* = 0.318, Fig. 5C; with medication/without a calling task, rs = -0.027, *p* = 0.912, Fig. 5D). In the context of an emergency event without medication and with/without a calling task, the smallest distance from the participant’s vehicle to pedestrian showed no correlation with the ratio of eye movements (without medication/without a calling task, rs = 0.186, *p* = 0.446, Fig. 6A; without medication/with a calling task, rs = 0.139, *p* = 0.572, Fig. 6B). Similarly, even when with medication and without a calling task, no correlation was observed (rs = 0.246, *p* = 0.311, Fig. 6C). However, under the combined influence of taking second-generation antihistamines and a calling task, a correlation emerged between the smallest distance from the participant’s vehicle to pedestrian and the ratio of eye movements (rs = 0.646, *p* = 0.0028, Fig. 6D).

**Fig. 7:**
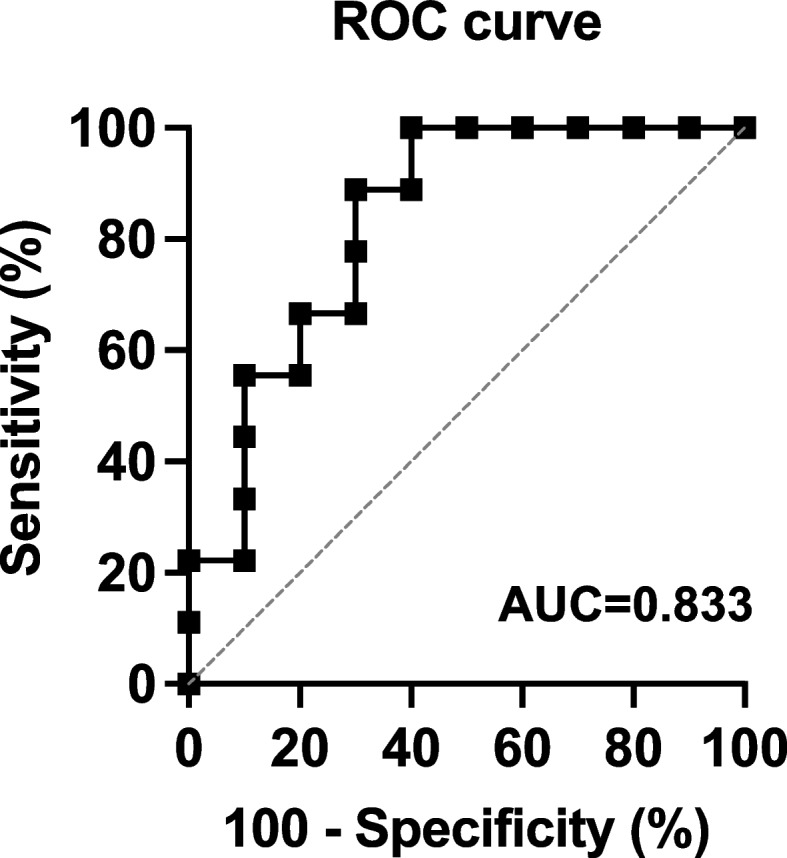
ROC analysis assessing the usefulness of eye movement as a discriminant index for the potential risk of reduced driving performance

Further analysis of the scatter plot in Fig. 6D revealed two distinct groups based on the proximity of the vehicle to the pedestrian. Consequently, the nine participants with shorter pedestrian distances were mean ± SD = 2.64 ± 0.33, while the ten participants with longer distances were mean ± SD = 12.2 ± 0.28. Since the subjects droved at 40 km/h (= about 11 m/s), there was about a 1-s difference in braking response between the two groups, meaning that the group with the shorter pedestrian distances was more likely to have an accident. Therefore, the nine participants with shorter pedestrian distances were categorized as the “dangerous driving group”, while the ten participants with longer distances were categorized as the “safe driving group. ROC analysis was performed to examine the usefulness of eye movements as a discriminative index of potential risk of reduced driving performance, yielding a high discriminative power with an AUC of 0.833 (Fig. 7).

## Discussion

Previous research reported a potential risk to driving performance during an emergency event when combining the use of second-generation antihistamines with a calling task [5]. This study aimed to investigate the correlation between driving performance and eye movements under these combined conditions, assessing the usefulness of eye movement measurements as a discriminative index for detecting potential risks of driving performance impairment.

The findings revealed a correlation between diminished driving performance and a reduced ratio of eye movements only during an emergency event that involved both the taking of second-generation antihistamines and performing a calling task. Previous studies have indicated that hands-free calling can narrow a driver’s visual field [12], and the impairment in driving performance associated with hands-free calling has been attributed, in part, a reduction in attention to eye input [13]. Histamine plays a vital role in sustaining vigilance and attention [14, 15], especially during tasks that require divided attention [16]. While second-generation antihistamines are recognized for having fewer central nervous system effects compared to their first-generation counterparts, blocking histamine receptors in the brain can still lead to reduced vigilance and attention, potentially contributing to the observed decrease in eye movements [3]. Therefore, it is posited that under the dual influence of second-generation antihistamines and a calling task, the observed decline in driving performance is associated with a decrease in the ratio of eye movements.

Furthermore, the results revealed a distinct division among participants into two groups based on their driving performance: nine participants were identified within the dangerous driving group due to their shorter distances from their vehicle to the pedestrian, while ten participants were classified within the safe driving group, maintaining longer distances. To assess the usefulness of eye movement as a discriminant index of driving performance risk, ROC analysis was conducted. ROC analysis is a widely accepted method across various fields for evaluating the discriminative capability of a diagnostic test [17]. The AUC obtained from ROC analysis serves as a crucial metric for gauging the efficacy of diagnostic methods, with an AUC of 0.7 or above generally indicating a useful discriminative index [18, 19]. Although an AUC exceeding 0.7 does not guarantee utility in every context, contingent upon the dataset size and study nature, the AUC of 0.833 in this research suggests the usefulness of eye movement measurements as a discriminative index for detecting potential risks of driving performance impairment.

A limitation of this study is its reliance on a driving simulator's virtual environment, which may not fully capture the complexities of real-world driving [20, 21]. Conducting experiments involving emergency scenarios in actual driving conditions poses significant safety risks, making simulators a necessary alternative. Despite potential discrepancies between simulator-based and real-world driving outcomes, the correlation between these results under specific conditions suggests the validity of the simulator findings in reflecting real-world driving behaviors [14, 22, 23]. Additionally, despite all participants undergoing two days of pre-test training to operate the driving simulator and respond to one-back tasks identical to those in the main tests, the potential confounding effects of learning cannot be completely ruled out. Therefore, further research is required to ascertain whether the results obtained in this small-scale study are replicable in larger-scale studies with randomized experimental conditions on a driving simulator. The final technical implementation of this research is a driving assistance technology that alerts the driver in advance to the risk of accidents due to the effects of medication and subtasks, by recording only the individual's eye movement measurements while driving in a real car. As a first step in this research, a large driving simulator measurement system was used. It is also necessary to accumulate more patient data for the final technical implementation. Therefore, as a second step, the aim is to develop a system that enables measurement in places such as community pharmacies, where there are no such driving simulator facilities. Based on the results of the present study, a simplified driving simulator and eye measurement environment that enables the determination of driving risk in community pharmacies will be developed and a demonstration study will be conducted. This development will enable a wider range of patients to be targeted than ever before. The introduction of this simplified driving simulator anticipates the possibility of being able to determine in advance the driving risk of patients due to complex conditions such as subtasks and medication other than second-generation antihistamines. Visualize potential driving risks for a larger number of patients and providing on-the-spot driving risk advice to patients from pharmacy pharmacists may be effective in preventing accidents.

## Conclusion

Eye movement measurement suggests potential risks of driving performance impairment. The findings of this study show the usefulness of driver's eye movement measurement to detect potential risks under the concurrent influence of second-generation antihistamines and a calling task.

## Data Availability

All data generated or analyzed during this study are included in this published article.

## Abbreviations

ROC: Receiver operating characteristic
AUC: Area under the curve
